# Biodegradable Food Packaging Films Using a Combination of Hemicellulose and Cellulose Derivatives †

**DOI:** 10.3390/polym16223171

**Published:** 2024-11-14

**Authors:** Syed Ammar Hussain, Madhav P. Yadav, Brajendra K. Sharma, Phoebe X. Qi, Tony Z. Jin

**Affiliations:** Eastern Regional Research Center, Agricultural Research Service, U.S. Department of Agriculture, 600 E. Mermaid Lane, Wyndmoor, PA 19038, USA; syed.a.hussain@usda.gov (S.A.H.); madhav.yadav@usda.gov (M.P.Y.); brajendra.sharma@usda.gov (B.K.S.); phoebe.qi@gmail.com (P.X.Q.)

**Keywords:** biobased films, hemicellulose, methyl cellulose, plasticizers, physiochemical properties, sustainable packaging

## Abstract

This study aims to develop biodegradable films by combining hemicellulose B (HB) with methylcellulose (MC) and carboxymethyl cellulose (CMC) at two mass ratios, HB/MC 90/10 and HB/CMC 60/40. The effect of plasticizers, glycerol (GLY) and polyethylene glycol (PEG), on these films’ mechanical and physicochemical properties was also investigated. Results showed that the film thickness increased with the addition of GLY and PEG. Moisture content was lower in plasticized films, possibly contributing to better storage. Plasticizers also induced more pronounced color changes, intensifying the lightness and yellowness. Physical attributes such as peel ability, foldability, and transparency were also noticeably improved, particularly in films with higher GLY and PEG concentrations. Additionally, plasticizers enhanced the mechanical properties more significantly in the HB/CMC films, as evidenced by improved tensile stress, elongation at break, elastic modulus, and toughness. However, oxygen and water vapor permeabilities, two of the most critical factors in food packaging, were reduced in the HB/MC films with plasticizers compared to the HB/CMC counterparts. The findings of this study bear significant implications for developing sustainable packaging solutions using hemicellulose B isolated from agricultural material processing waste. These biopolymer-based films, in conjunction with biobased plasticizers, such as glycerol biopolymer, can help curtail our reliance on conventional plastics and alleviate the environmental impact of plastic waste.

## 1. Introduction

Polymers play a crucial role in modern life due to their ease of production and wide range of functional properties. In 2017, global plastic production, including thermoplastics, thermosets, elastomers, adhesives, coatings, sealants, and PP-fibers, was around 348 million tons, increasing to 359 million tons in 2018. Asia, particularly China, is a principal contributor to this production, with Europe, the Middle East, and Africa also playing significant roles [1]. India is a leading producer and consumer of plastics, especially polyethylene (PE), commonly used to manufacture packaging materials, including films and sheets. In just two years, 2018 and 2019, India produced over 15 million tons of plastic and is projected to reach 24 million tons by 2020 [2].

Most plastics (95–99%) are derived from non-renewable petrochemical sources [3]. These synthetic plastics are extensively used in the medical, packaging, and construction sectors. In India, 43% of synthetic polymers produced annually are used in packaging. However, synthetic plastics do not degrade physically, chemically, or biologically, leading to significant environmental [3,4,5,6,7,8,9,10] and health issues [11]. They eventually turn into microplastics that have been detected nearly everywhere, including underground water resources, fishes, animals, and humans. Accumulated waste clogs drainage systems and harms aquatic life, and incineration releases harmful gases, contributing to air pollution and global warming [12]. The persistence of synthetic polymers has raised growing global concerns and is driving the search for eco-friendly alternatives [13].

Biodegradable polymers have emerged as a promising solution for a variety of industrial applications and hold the prospect of mitigating the environmental risks associated with non-biodegradable plastics [14,15,16,17,18,19,20]. They have also demonstrated significant potential in food packaging applications, providing desirable barrier properties and mechanical strength [21,22,23,24,25,26]. Among biodegradable polymers, hemicellulose, a polysaccharide derived from plant cell walls, has gained attention due to its abundance and favorable properties. Xylan, a major component of hemicellulose, is particularly noteworthy, as it constitutes a significant portion (40–45% of dry weight) of plant biomass and possesses flexible properties that mimic petroleum-based plastics. This makes them a viable candidate for developing eco-friendly packaging materials [27,28].

Despite the immense potential, the application of hemicellulose-based films in packaging is limited by certain drawbacks, such as hygroscopicity, brittleness, and inferior mechanical properties [29,30]. To overcome these challenges, the scientific research community has focused on enhancing the properties of hemicellulose films by incorporating additional hardeners like methyl cellulose (MC) and carboxymethyl cellulose (CMC). MC and CMC are cellulose derivatives known for their excellent film-forming capabilities and compatibility with other materials [30,31,32,33,34,35]. Combining hemicellulose with these derivatives can produce films possessing enhanced barrier properties and mechanical strength, making them ideal for food packaging. Plasticizers can further reinforce hemicellulose-based films, leading to better outcomes in developing biodegradable food packaging alternatives.

The agricultural processing by-products (lignocellulosic materials) mainly comprise cellulose, hemicellulose, and lignin [36,37]. Hemicellulose, which comes second to cellulose in its abundance, has shown a broad application prospect due to its extensive sources, renewability, and biodegradability. Unlike cellulose, a homogeneous glycan structure composed entirely of β-(1→4)-glucan connecting to the dextran chain, hemicellulose from agricultural material is composed of β-(1→4) linked xylan backbone with arabinose and a small amount of galactose, glucose, glucuronic acid, and galacturonic acid in the side, making its highly branched structure. The most abundant hemicelluloses are arabinoxylan, xylan (annual plants and hardwoods), and mannans (softwoods). Hemicelluloses are concentrated in the agricultural by-products, which offer an opportunity to develop value-added products. Although hemicelluloses constitute about 40–45% of the dry weight of annual plants (crops), they have thus far not been exploited industrially. Corn brans are by-products of the corn-milling industry for ethanol [38]. It has little economic value and frequently becomes a waste disposal problem [39]. Large quantities of this agricultural processing by-product are a low-cost feedstock that can be processed into a value-added product like arabinoxylan (HB).

Our group has successfully developed a patented method for separating high-value arabinoxylan and cellulose-rich fractions from many grains and agricultural processing by-products, energy crops, and agricultural residues [40,41,42]. We have studied the applications of corn arabinoxylans as emulsifiers, healthy dietary fiber with antioxidant activity, binder for briquette [40,43], and viscosity modifier [44]. Now, we are exploring the potential of this material to fabricate high-quality biodegradable packaging films. Arabinoxylans separated from other feedstocks, such as sorghum bran, bagasse, and biomass, have been studied in making films and shown to have sensitivity to moisture adsorption and favorable strength when using glycerol as a plasticizer [45].

In this preliminary study, we combined our lab-produced HB from corn bran, also termed “corn bio-fiber gum” (corn BFG), with the commercial carboxymethyl cellulose (CMC) and methyl cellulose (MC) to form the biopolymer bases and evaluated their film-forming ability. In future studies, we plan to prepare the cellulose-rich fractions (CRFs) from agricultural biomass and derivatize CRFs to produce carboxymethyl and methyl derivatives in the lab. We expect that in such a polymer blend, the CRF derivatives will be evenly distributed into the hemicellulose matrix, changing the microstructure and composition of hemicellulose film through the action of non-chemical bonds and thus improving its mechanical properties. Including cellulose derivatives in HB films will improve the mechanical and barrier properties and transparency and help pave the way to utilize the CRF by-product generated in the process to maximize carbon utilization efficiency.

Biodiesel production has been growing steadily worldwide [46], and the demand for renewable fuel is increasing to lower the GHG potential of this manufacturing. This has resulted in a large surplus of glycerol, a by-product of the biodiesel industry. Valorizing this by-product is attractive and presents the biodiesel industry as a viable and competitive option. In addition to glycerol, we studied another biobased plasticizer, sorbitol, and two polyethylene glycols (PEGs) of different molecular weights (300 and 1000). Of these, we selected the best-performing ones, glycerol (GLY) and two types of polyethylene glycol (PEG 300 and PEG 1000), for further study. Therefore, this study aimed to develop biobased packaging films by combining hemicellulose and cellulose derivatives with acceptable physiochemical and mechanical properties and investigate how these two plasticizers could enhance the properties of the films.

## 2. Materials and Methods

### 2.1. Materials

Carboxymethyl cellulose (CMC), methylcellulose (MC), polyethylene glycol (PEG 300 and PEG 1000), and glycerol were purchased from Millipore Sigma (St. Louis, MO, USA). Hemicellulose B (HB) was prepared in our lab (see detail below). Deionized water was obtained from the Milli-Q Advantage A10 ultrapure water purification system. All chemicals were reagent grade.

### 2.2. Preparation of Hemicellulose B (HB)

Hemicellulose B (HB) was extracted from corn bran using a modified version of the previously published methods [43,47,48]. Initially, ground and de-oiled corn bran was suspended in water and boiled at 85 °C with a pH of 6.80 in the presence of α-amylase for one hour. The pH was then adjusted to 11.5 by adding 50% NaOH, and the hot reaction mixture was stirred for another half an hour. The hot slurry of the deconstructed corn bran was sheared at 10,000 rpm for 30 min and then cooled to room temperature. The mixture was centrifuged at 14,000× *g* for 10 min, and the supernatant was collected. The pH of the supernatant was adjusted to about 4 to precipitate HA, which was collected by centrifugation. The supernatant obtained from the HA collection was used for HB preparation. To precipitate the hemicellulose B fraction, two volumes of ethanol were gradually added to the supernatant obtained after HA separation, with continuous stirring. The precipitated HB was filtered, washed with 100% ethanol three times to obtain pure HB, and dried in a vacuum oven at 50 °C. The purity of HB was confirmed by a high-performance size exclusion chromatography (HPSEC) system, which was connected to a multi-angle laser light scattering photometer (MALLS) (Wyatt Technology, Santa Barbara, CA, USA) and RI detectors [40].

### 2.3. Preparation of HB/MC 90/10 and HB/CMC 60/40 Films

To fabricate the films, the HB, MC, and CMC solutions were prepared separately in deionized water by adding their calculated amount (3.33%) and mixing overnight until a fully homogeneous solution was obtained. The solutions were combined in the 90:10, 80:20, 70:30, and 60:40 (HB: MC/CMC) ratios, stirred, and degassed overnight under a house vacuum (0.1 atm, 25 °C). The resulting mixtures were poured into 100 mm Teflon Petri dishes (30.0 g each) (Welch Fluorocarbon, Inc., Dover, NH, USA) and allowed to dry in an environmental chamber (Model 7900-33, Caron Scientific, Marietta, OH, USA); they were dried (at (20 °C and 50% RH)) for about a week until a consistent weight of 1.11 g was achieved. The dried films were peeled off and evaluated for strength and flexibility. An initial screening (based on these physical attributes) revealed that 90:10 (HB/MC) and 60:40 (HB/CMC) mass ratios performed the best. For plasticized films, the respective HB/MC or HB/CMC solutions were divided into ten parts in ten different flasks, with eight containing plasticizers (GLY or PEG) at 15% and 20% (*w*/*w*) levels and two without plasticizer serving as a control. Each solution was homogenized by stirring, degassed, poured into Teflon Petri dishes, dried, and assessed similarly. The dried films were stored in Ziplock bags in desiccators for further testing.

### 2.4. Film Characterization

#### 2.4.1. Physical Attributes

A scoring system from −2 to 2 was used to quantify the results of peel ability, foldability, transparency, and the appearance of air bubbles. Each film was scored based on the ease of peeling from a Petri dish. A score of −2 was given if the film could not peel from the Petri dish or it broke as the test was completed. Each film was folded softly at a bilateral angle to determine the foldability of the film. Complete breakage resulted in a −2, and no visible breakage resulted in a 2. Each film was placed up against a sign with black letters. The unclear appearance of the sign resulted in a score of −2. Complete visual transparency was assigned a score of 2. Each film was examined for the presence of air bubbles. A score of −2 was given if the film had many air bubbles present. A score of 2 was given if the film had no presence of air bubbles. The total score of all attributes was 8.

#### 2.4.2. Colorimetry

The digital colorimeter (PCE-CSM 1, PCE Americas Inc., Jupiter, FL, USA) was used to measure the hunter LAB properties of the film: L* representing the whiteness, a* representing the redness/greenness, and b* representing the yellowness/blueness of each film. Films were placed on a sheet of standard white paper with L reference = 94.48, a reference = 0.41, and b reference = 0.03, and data were collected at three random spots. The collected data were used to calculate the whiteness index (WI), yellow index (YI), and total color difference (TCD), as described previously [49].

#### 2.4.3. Film Thickness and Moisture

Film thickness (µm) was calculated using a 0–1″/0–25 mm Xtra-Value II Electronic micrometer (Fowler High Precision, Canton, MA, USA). Each sample was taken in triplicate for all films, where average values were calculated and reported. For the moisture content measurement, each film was placed in a Moisture Analyzer (Torbal ATS 133, Scientific Industries, Inc., Bohemia, NY, USA) at 120 °C until the end of the process, notified by the machine. The initial and end weights were recorded. The moisture percentage was calculated using the instrument’s software.

### 2.5. Physiochemical Properties of Films

#### 2.5.1. Oxygen Transmission

The oxygen transmission rate (OTR) of the films was measured using the OX-TRAN Model 1/50 (MOCON, Minneapolis, MN, USA), following the standard method ASTM D3985. The film samples were conditioned and mounted as a sealed barrier between two chambers. One chamber was purged with nitrogen (carrier gas), while the other contained oxygen (test gas). Oxygen permeated through the film into the nitrogen stream then was transported to a coulometric detector. The detector measured the amount of oxygen passing through the film per unit time. The OTR results were expressed in cubic centimeters per square meter per day (cc/m^2^/day) at 23 °C and 0% relative humidity (RH).

#### 2.5.2. Water Vapor Permeability

The water vapor transmission rate (WVTR) of the films was measured using the PERMATRAN-W Model (MOCON, Minneapolis, MN, USA), following the standard method ASTM E96/E96M. The film samples were mounted in a test cell. One side of the film was exposed to a humidity-controlled environment, while the other side was exposed to a dry condition. The amount of water vapor passing through the film was measured over time. The WVTR results were expressed in grams per square meter per day (g/m^2^/day) at 23 °C and 50% RH.

### 2.6. Mechanical Properties

The mechanical properties of the films were determined according to the standard method ASTM D882. Films were cut into strips (20 mm × 40 mm) and placed in a desiccator for 48 h at 22 °C and 50% relative humidity (RH) by using saturated potassium chloride (KCl) solution. Tests were performed in Texture analyzer TA. XT+ (Stable Micro Systems, Godalming, Surrey, UK). The film strip’s initial length was set to 21 mm then stretched at a constant velocity of 2 mm/min until reaching a breaking point. The stress–strain curves were computer-recorded by the software Exponent (version 2.64), and other mechanical properties were calculated based on these curves [22,47].

### 2.7. Statistical Analysis

All experiments were replicated three times, and data were reported as mean  ±  standard deviation. One-way ANOVA with post hoc Turkey’s test was conducted using the GraphPad software (GraphPad Prism 7.0 USA).

## 3. Results and Discussion

Our initial testing found that the glycerol level affects the mechanical properties of the films. Therefore, we chose two plasticizer concentrations, 15% and 20%. Furthermore, we analyzed and assessed 36 biofilms incorporated with three different plasticizers, glycerol, sorbitol, and polyethylene glycols (PEG 300 and PEG 1000), based on their physicochemical properties. Subsequently, we narrowed down to 10 best-performing films, i.e., HB/MC 90/10, HB/MC + 15% glycerol, HB/MC + 20% glycerol, HB/MC + 15% PEG 1000, HB/MC + 20% PEG 1000, HB/CMC 60/40, HB/CMC + 15% glycerol, HB/CMC + 20% glycerol, HB/CMC + 15% PEG 300, and HB/CMC + 20% PEG 300, for further investigation, as documented in this paper.

### 3.1. Physical Attributes

**Figure 1:**
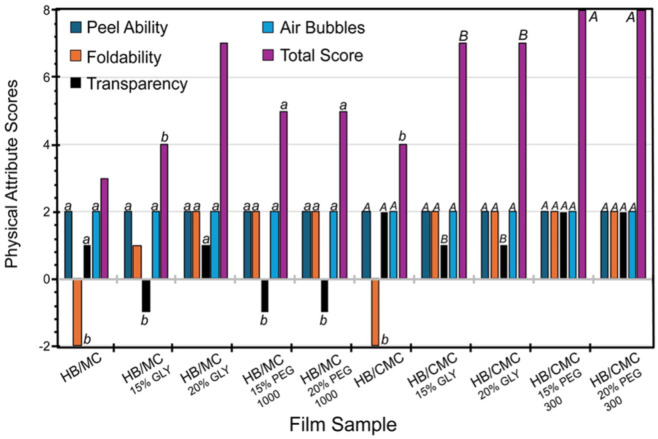
Physical attributes of HB/MC 90/10 and HB/CMC 60/40 films. Data are mean  ±  standard deviation (*n*  =  3). Data sharing the same letter are not statistically significantly different (*p*  >  0.05).

Figure 1 shows the physical attributes of the films scored as described above. For the HB/MC 90/10 films, adding plasticizers improved peel ability, foldability, and transparency. Notably, the film containing 20% glycerol achieved the highest total score of 7 (Figure 1). In the case of HB/CMC 60/40 films, incorporating glycerol and PEG 300 at varying percentages positively impacted all physical attributes, particularly enhancing foldability compared to the control. Films with PEG 300 showed a maximum total score of 8 (Figure 1). These findings highlighted the importance of plasticizers in optimizing film properties for potential applications.

The physical attributes of the films, including peel ability, foldability, and transparency, were significantly improved with the addition of plasticizers. Notably, the HB/MC 90/10 films containing 20% glycerol achieved the highest total score for these attributes. The HB/CMC 60/40 films with varying percentages of glycerol and PEG 300 also showed enhanced foldability and overall usability.

### 3.2. Film Color

Table 1 shows the color analysis of the films studied. For the HB/MC 90/10 films, the control film (HB/MC) exhibited moderate lightness (L = 86.29) and a slightly yellowish hue (a = 2.34, b = 15.60). When 15% glycerol was added, the film became more yellow (yellow index: 28.96) and showed an overall color difference of 112.35 (TCD). The 20% glycerol film maintained similar lightness but showed a more pronounced yellow tint (b = 17.99). Films with 15% PEG 1000 had lower lightness (L = 84.73) and higher yellowness (yellow index: 30.98). Notably, the 20% PEG 1000 film presented the highest color difference (TCD was 132.91), indicating significant color alteration compared to the control film (Table 1).

For the HB/CMC 60/40 series (Table 1), the control film (HB/CMC) exhibited moderate lightness (L = 86.09) and a slightly yellowish hue (a = 1.181, b = 11.65). When 15% glycerol was used, the film became more yellow (yellow index: 18.98) and showed a reduced overall color difference (TCD) of 64.10. The 20% glycerol film retained similar lightness but displayed a more pronounced yellow tint (b = 11.72). Films containing 15% PEG 300 had lower lightness (L = 87.66) and slightly higher yellowness (yellow index: 19.15). Interestingly, the 20% PEG 300 film displayed a higher color difference of 68.58 (TCD). These results highlighted the impact of plasticizers on the films’ color and whiteness, providing valuable insights for further analysis.

Results from the color analysis revealed that plasticizers significantly changed the films’ lightness and yellowness. For instance, the HB/MC 90/10 films with 15% and 20% glycerol exhibited more pronounced yellow tints and higher total color differences than the control. Similarly, the HB/CMC 60/40 films showed variations in lightness and yellowness, with plasticizers leading to noticeable color alterations. These color changes are notable considerations for the films’ aesthetic appeal in consumer applications [49].

### 3.3. Film Thickness and Moisture Content

**Figure 2:**
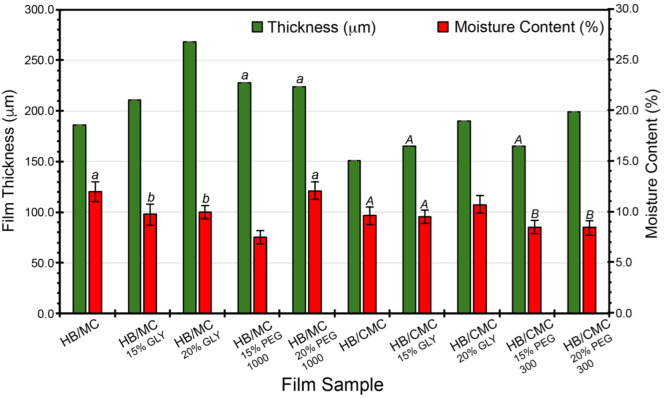
Thickness and moisture content (%) of HB/MC 90/10 and HB/CMC 60/40 films. Data are mean  ±  standard deviation (*n*  =  3). Data sharing the same letter are not statistically significantly different (*p* > 0.05).

Figure 2 shows the films’ thickness and moisture content measurements. The plasticizer increased the film thickness in the HB/MC 90/10 films, ranging from 211 to 268 µm, approximately 13% to 44%, compared to the control film thickness of 186 µm, shown in Figure 2. Similarly, including plasticizers impacted the thickness of the HB/CMC 60/40 films. The thickness of these films ranged from 165 µm to 199 µm, with a percentage increase from the control film thickness (165 µm) ranging from 0.01% to 20%.

The results showed a marked increase in film thickness with higher plasticizer concentrations, regardless of the type of plasticizer used. This effect is likely due to plasticizers disrupting and reorganizing the intermolecular polymer chain networks, resulting in more free volume and, thus, thicker films. Previous investigations reported similar observations on the impact of plasticizer concentration on film thickness [50,51,52,53,54,55,56].

Regarding the moisture content, the HB/MC 90/10 films containing 15% glycerol and 15% PEG 1000 exhibited lower moisture levels than the control. For the HB/CMC 60/40 films, those containing PEG 300 (at 15% and 20%) also showed reduced moisture content than the control film (Figure 2). PEG-plasticized films showed lower moisture content than glycerol-plasticized films, which is due to the lowered molecular weight of glycerol and is more hygroscopic than PEGs. Incorporating GLY and PEG into the biopolymer films produced a more flexible and less dense polymer matrix. This structural modification diminishes the film’s ability to absorb and retain moisture, as the free hydroxyl groups that typically attract water molecules are either occupied by hydrogen bonds with the plasticizers or are less accessible due to the increased free volume. Consequently, the reduced moisture content in the films lowers the risk of microbial growth and spoilage, thereby enhancing the protective qualities of the films [57].

### 3.4. Physiochemical Properties of Films

#### 3.4.1. Oxygen Transmission

**Figure 3:**
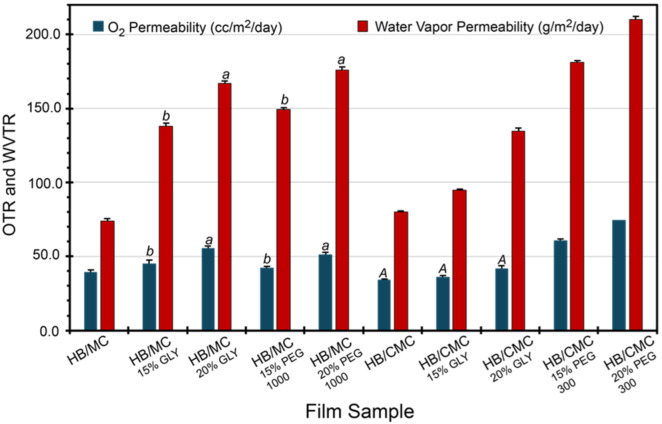
Oxygen and water vapor permeability of HB/MC 90/10 and HB/CMC 60/40 films. Data are mean  ±  standard deviation (*n *= 3). Data sharing the same letter are not statistically significantly different (*p* > 0.05).

Oxygen permeability is a crucial parameter in evaluating the effectiveness of food packaging materials. When environmental oxygen penetrates the packaging material, it can cause fatty acid oxidation in the packaged food, leading to quality deterioration and a shorter shelf life. Figure 3 provides detailed oxygen transmission profiles for the film samples. Notably, films containing HB/MC with varying plasticizer types and concentrations exhibit higher oxygen permeability than the control. Likewise, plasticizer types and levels also elevated the oxygen permeability of HB/CMC 60/40 films compared to the control samples (Figure 3).

Although water-soluble plasticizers improve the mechanical properties, they affect the barrier properties, as seen in our films. An earlier study [28] showed that the plasticizer concentration below 10% improves the water vapor permeability but negatively affects it at a higher than 10% concentration. Higher plasticizer concentrations mean excess plasticizers, generating more affinity to water. More affinitive water molecules break chain-to-chain interactions, introducing more free volume and causing higher water vapor permeability. Figure 3 also suggests that plasticizers negatively influenced the oxygen permeability of both HB/MC and HB/CMC films compared to the control films studied in this work. This change is attributed to the addition of plasticizers, which reduces hydrogen bonding between polymer chains, making the structure less dense and more flexible. This increases free volume by disrupting the tight packing of polymer chains, allowing oxygen molecules to move more freely.

Additionally, plasticizers enhance the mobility of polymer chains, facilitating the diffusion of oxygen molecules. These changes collectively increased oxygen permeability when plasticizers were added to polymers [58]. Nevertheless, glycerol-containing films showed exceptional oxygen permeability values, consistent with previous studies on MC and MC/beeswax composite films [59,60].

Even though the oxygen permeability of our HB/MC and HB/CMC films was higher due to the inclusion of plasticizers, it still fell within an acceptable range for some food packaging applications. Compared to commercial biodegradable films [18,61,62], our films exhibit comparable or slightly higher permeability values, which can be mitigated using additional barriers or coatings, such as nitrocellulose lacquer, to improve cellophane films’ permeability. Despite this, the films offer significant environmental benefits by utilizing agricultural by-products, contributing to waste reduction, and promoting sustainability. A trade-off between slightly higher permeability and environmental advantages makes these films a viable option for eco-friendly packaging solutions. Furthermore, the cost-effectiveness and availability of the raw materials used in our films enhance their practicality for real-world applications, aligning with circular economy principles and supporting the transition to more sustainable packaging practices.

#### 3.4.2. Water Vapor Permeability (WVP)

The films’ water vapor permeability (WVP) varied between 73 and 210 gm/m^2/day (Figure 3), depending on the film composition, plasticizer type, and concentration. Methylcellulose (MC)-based films are more hydrophilic than carboxymethyl cellulose (CMC), causing the lowered WVP in the CMC-based films. This difference might be attributed to the three-dimensional dense structures of the CMC-based films. The incorporation of plasticizer increased the WVP of all films. Our results are consistent with previous studies speculating that incorporating plasticizers can create hydrogen bonds with hydrophilic parts of the polymers, reducing the associated free volume. As a result, plasticizer helps increase the mobility of polymer chains and allows for greater diffusion of water molecules through the film matrix [63,64,65].

#### 3.4.3. Mechanical Properties

The films exhibited improved mechanical properties when plasticizers (GLY and PEG) were used, including tensile stress, elongation, elastic modulus, and toughness. These plasticizer molecules likely inserted themselves between the HB and MC/CMC chains, disrupting the existing hydrogen bonds and creating new interactions with the hydroxyl groups of the plasticizers. This disruption increased the free volume within the polymer matrix, making the films more flexible and less brittle. However, the interaction between HB and MC was relatively weak due to MC’s limited hydrogen bonding capacity, which may explain the moderate improvements seen in the HB/MC films’ mechanical properties [66,67].

**Figure 4:**
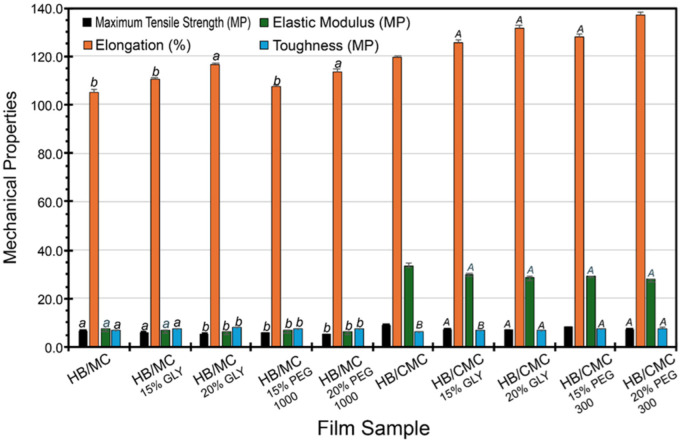
Mechanical strength for HB/MC 90/10 and HB/CMC 60/40 films. Data are mean  ±  standard deviation (*n*  =  3). Data sharing the same letter are not statistically significantly different (*p* > 0.05).

In comparison, the incorporation of plasticizers into HB/CMC films led to significant structural changes in the film matrix. The plasticizers disrupted the hydrogen bonds between HB and CMC, but the carboxymethyl groups in CMC facilitated the formation of new hydrogen bonds with the plasticizers. This resulted in a more flexible and cohesive polymer network, enhancing mechanical properties such as tensile strength, elongation, and toughness. The higher compatibility between HB and CMC due to these strong intermolecular hydrogen bonds was evident in the superior mechanical performance of the HB/CMC films. The HB/CMC films exhibited more pronounced improvements in mechanical properties than the HB/MC films (Figure 4). In our study, the highest elongation at break was 137.17% for film HB/CMC (with 20% PEG), which exceeded previous investigations such as 64% for sugarcane bagasse [68,69,70,71,72,73]. However, the maximum tensile strength in our aforesaid blend films was 10.45 MP, which was much higher than curaura fiber-based films (2.22 MP) [71] and comparable to wheat straw-based films (12.00 MP) [72]. Overall, our blend films showed a 10–65% increase in tensile strength when compared to the control films. This trend can be attributed to the stronger intermolecular hydrogen bonding between HB and CMC and cross-linking potential, further stabilized by the plasticizers [66,67] and underscoring the importance of the chemical compatibility between the biopolymers and the plasticizers [74,75]. Increasing the plasticizer concentration also showed a consistent increase in elongation for all cases. 

Moreover, a previous study [74] indicated that in biomacromolecules, mechanical strength depends primarily on the formation and stability of hydrogen bonds within their structures. In sum, our results were consistent with previous investigations, which have shown that plasticizers can decrease tensile stress and Young’s modulus while increasing percent elongation, toughness, and resistance to cracking, thereby improving the overall mechanical performance of the polymer [76,77].

Despite these promising results, this study has some limitations. The films’ water vapor permeability (WVP) suggests that additional hydrophobic functionality is needed to enhance this property. While high plasticizer concentrations reduce film brittleness, targeting a lower plasticizer concentration (≈10%) could help decrease processing costs, provided that acceptable mechanical strength is maintained. Moreover, the long-term stability and biodegradability of the plasticized films were not assessed. Future studies should investigate these films’ environmental impact and degradation behavior over time [17,77,78,79,80,81,82,83,84,85,86,87,88,89]. The plasticized films’ inferior WVP and ambient enhanced physical and mechanical properties indicate much improvement needs to be considered and researched.

## 4. Conclusions

This study demonstrated that combining hemicellulose (HB), an agricultural by-product from the corn-milling process, and cellulose derivatives (MC and CMC) with appropriate plasticizers created food packaging films with excellent physical attributes and mechanical properties, paving the way for their potential application in sustainable packaging solutions. Despite their less-than-optimal water and oxygen permeability performance, these films hold immense economic and environmental promise. This research will continue to explore the use of other natural additives from agricultural processes to improve and refine these critical qualities. Additionally, further comprehensive testing of these films’ long-term stability, cost-effectiveness, and production scalability is necessary to validate these findings fully for broader applications.

## Figures and Tables

**Table 1:** Color analysis for HB/MC 90/10 and HB/CMC 60/40 films at various levels of plasticizers.

Film Composition	L*	a*	b*	Whiteness Index	Yellowness Index	Total Color Difference (TCD)
HB/MC 90/10	86.29 ± 0.16	2.34 ± 0.01	15.60 ± 0.02	79.10 ± 0.91	25.82 ± 1.01	92.70 ± 1.02
HB/MC + 15% GLY	85.36 ± 0.39 ^a^	2.59 ± 0.02 ^a^	17.30 ± 0.04 ^a^	77.19 ± 0.02 ^a^	28.96 ± 0.92 ^a^	112.35 ± 1.07 ^a^
HB/MC + 20% GLY	85.67 ± 0.21 ^a^	2.65 ± 0.01 ^a^	17.99 ± 0.16 ^a^	76.85 ± 0.44 ^ba^	30.00 ± 0.23 ^a^	107.55 ± 1.04 ^a^
HB/MC + 15% PEG 1000	84.73 ± 0.32 ^b^	2.96 ± 0.01	18.37 ± 0.05	75.92 ± 0.43 ^a^	30.98 ± 0.34 ^a^	127.67 ± 1.11
HB/MC + 20% PEG 1000	84.38 ± 0.42 ^b^	2.65 ± 0.02 ^a^	17.99 ± 0.11 ^a^	76.03 ± 0.33 ^a^	30.46 ± 0.11 ^a^	132.91 ± 1.21
HB/CMC 60/40	86.09 ± 0.09	1.181 ± 0.05	11.65 ± 0.19 ^A^	81.77 ± 1.08 ^A^	19.33 ± 1.15 ^A^	14.99 ± 0.07
HB/CMC + 15% GLY	87.68 ± 0.17 ^A^	1.49 ± 0.021	11.65 ± 0.15 ^A^	82.98 ± 1.12 ^A^	18.98 ± 1.2 ^A^	64.10 ± 1.32 ^A^
HB/CMC + 20% GLY	88.00 ± 0.18	1.56 ± 0.031 ^A^	11.72 ± 0.18 ^A^	83.15 ± 1.22 ^A^	19.03 ± 1.18 ^A^	59.91 ± 0.92
HB/CMC + 15% PEG 300	87.66 ± 0.20 ^A^	1.51 ± 0.032 ^A^	11.75 ± 0.11 ^A^	82.89 ± 1.31 ^A^	19.15 ± 1.22 ^A^	64.59 ± 0.33 ^A^
HB/CMC + 20% PEG 300	87.41 ± 0.26 ^A^	1.53 ± 0.032 ^A^	11.95 ± 0.12	82.57 ± 1.11 ^A^	19.54 ± 1.31 ^A^	68.58 ± 0.81

* Data are represented as means (*n*  =  3)  ±  standard deviations. Values sharing the same letter within the same film series (and in the same column) indicate that the differences are statistically insignificant (*p*  >  0.05).

## Data Availability

Data is contained within the article.
